# Functional interplay between cylindromatosis and histone deacetylase 6 in ciliary homeostasis revealed by phenotypic analysis of double knockout mice

**DOI:** 10.18632/oncotarget.8374

**Published:** 2016-03-25

**Authors:** Jie Ran, Fan Yu, Juan Qin, Yijun Zhang, Yunfan Yang, Dengwen Li, Jun Zhou, Min Liu

**Affiliations:** ^1^ Institute of Biomedical Sciences, College of Life Sciences, Key Laboratory of Animal Resistance of Shandong Province, Key Laboratory of Molecular and Nano Probes of the Ministry of Education, Shandong Normal University, Jinan 250014, China; ^2^ State Key Laboratory of Medicinal Chemical Biology, College of Life Sciences, Nankai University, Tianjin 300071, China

**Keywords:** cylindromatosis, histone deacetylase 6, knockout mouse, cilium, centrosome

## Abstract

Cilia are present in most vertebrate tissues with a wide variety of functions, and abnormalities of cilia are linked to numerous human disorders. However, the molecular events underlying ciliary homeostasis are poorly understood. In this study, we generated double knockout (DKO) mice for the deubiquitinase cylindromatosis (CYLD) and histone deacetylase 6 (HDAC6), two critical ciliary regulators. The *Cyld*/*Hdac6* DKO mice were phenotypically normal and showed no obvious variances in weight or behavior compared with their wild-type littermates. Strikingly, *Cyld* loss-induced ciliary defects in the testis, trachea, and kidney were abrogated in the *Cyld*/*Hdac6* DKO mice. In addition, the diminished α-tubulin acetylation and impaired sonic hedgehog signaling caused by loss of *Cyld* were largely restored by simultaneous deletion of *Hdac6*. We further found by immunofluorescence microscopy a colocalization of CYLD and HDAC6 at the centrosome/basal body and, interestingly, loss of *Cyld* promoted the localization of HDAC6 at the centrosome/basal body. These findings provide physiological insight into the ciliary role of the CYLD/HDAC6 axis and suggest a functional interplay between these two proteins in ciliary homeostasis.

## INTRODUCTION

Cilia extend from the surfaces of most mammalian cell types, functioning as sensory and motile organelles. A variety of developmental disorders and pathological conditions have been shown to arise from ciliary defects and are termed ciliopathies [1]. Mutations in cilium-associated structural or signaling proteins cause insensitivity to environmental signals, resulting in disorganized and hyperplastic cell growth in disorders such as polycystic kidney disease (PKD) and Bardet-Biedl syndrome (BBS). At the organismal level, ciliary defects induce infertility, respiratory diseases, renal cysts, *situs inversus*, and hypertension. Over the past decade, our understanding of the composition and structure of cilia have been greatly improved [2]. However, little is known regarding the mechanisms that regulate ciliary homeostasis.

Cylindromatosis (CYLD), a microtubule-associated deubiquitinase involved in the regulation of diverse biological processes such as cell signaling [3], cell migration [4–6], cell cycle progression [7–11], and cancer [12], has recently been identified as a crucial player in ciliogenesis [13, 14]. *Cyld* knockout (CKO) mice exhibit ciliary defects across multiple organs, varying from structural and functional deficiencies to cilium-related disorders [13]. Interestingly, inhibition of histone deacetylase 6 (HDAC6), a cytoplasmic enzyme critically involved in the regulation of cilia [15–27], with small-molecule inhibitors partially restores the ciliary defects in CKO mice [13]. To explore the physiological mechanisms underlying the ciliary role of the CYLD/HDAC6 axis, we generated *Cyld*/*Hdac6* double knockout (DKO) mice. Phenotypic characterization of these mice demonstrates a functional interplay between CYLD and HDAC6 in ciliary homeostasis.

## RESULTS

### Generation and confirmation of *Cyld*/*Hdac6* DKO mice

**Figure 1:**
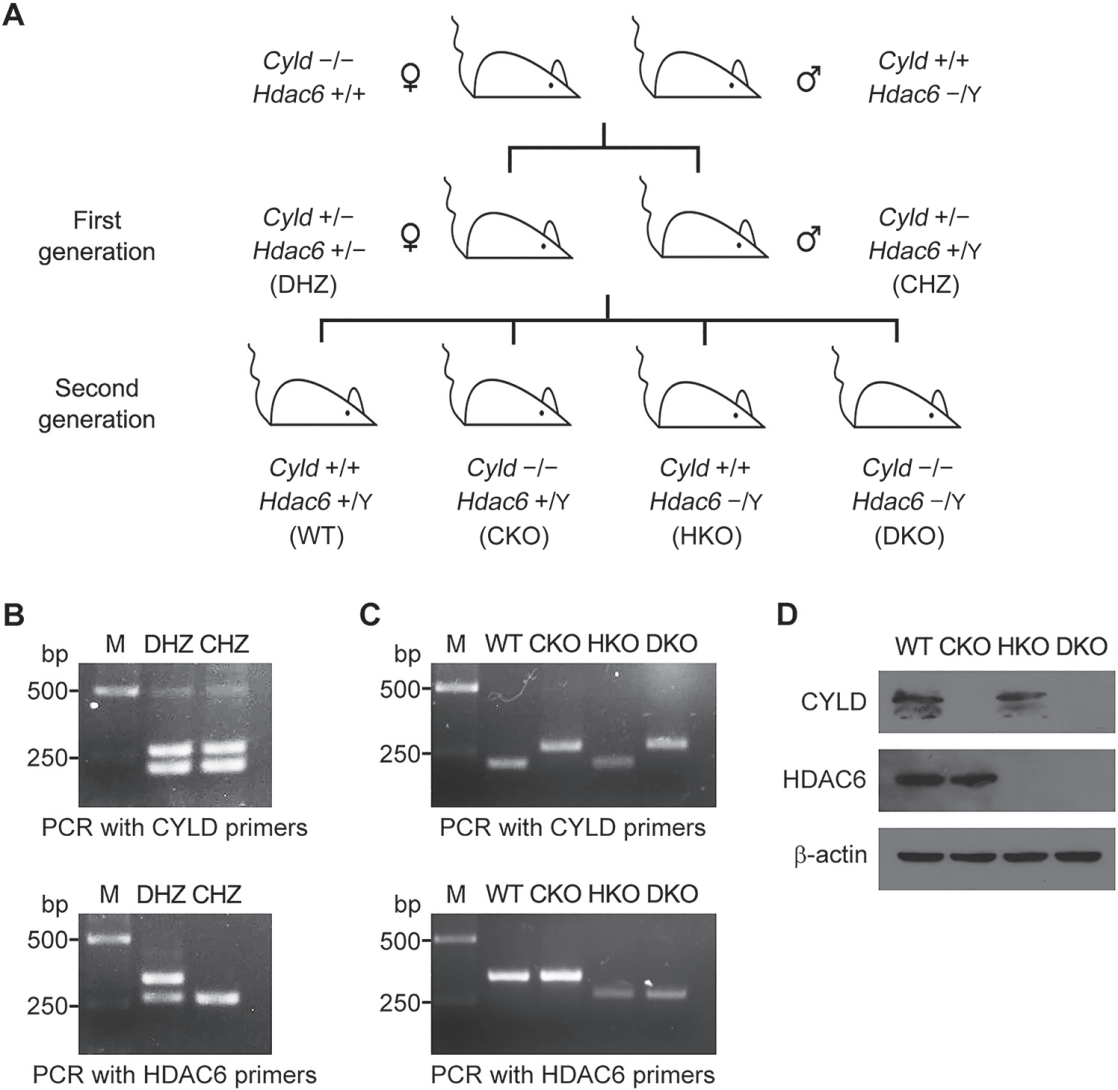
Generation and confirmation of *Cyld*/*Hdac6* double knockout (DKO) mice **A.** Protocol used for the generation of male *Cyld*/*Hdac6* DKO mice (i.e., *Cyld* −/−, *Hdac6* -/Y). **B.** Genotyping by PCR with *Cyld* and *Hdac6* primers to identify the first-generation mice. PCR was performed using mouse tail DNA from female *Cyld*/*Hdac6* double heterozygous (DHZ) mice (i.e., *Cyld* +/−, *Hdac6* +/−) and male *Cyld* heterozygous (CHZ) mice (i.e., *Cyld* +/−, *Hdac6* +/Y). **C.** Genotyping by PCR with *Cyld* and *Hdac6* primers to identify mice of the second generation. PCR was performed using tail DNA from male wild-type (WT) mice (i.e., *Cyld* +/+, *Hdac6* +/Y), male *Cyld* knockout (CKO) mice (i.e., *Cyld* −/−, *Hdac6* +/Y), male *Hdac6* knockout (HKO) mice (i.e., *Cyld* +/+, *Hdac6* -/Y), and male *Cyld*/*Hdac6* DKO mice. **D.** Western blot analysis of CYLD, HDAC6, and β-actin in the livers of WT, CKO, HKO, and DKO mice.

Because male CKO mice are infertile [28], and *Hdac6* is an X-linked gene [29], we selected female CKO mice (i.e., *Cyld* −/−, *Hdac6* +/+) and male *Hdac6* KO (HKO) mice (i.e., *Cyld* +/+, *Hdac6* −/Y) for the production of first-generation heterozygous mice (Figure 1A). Male *Cyld*/*Hdac6* DKO mice were then generated at a Mendelian frequency of 1:16 in the second generation by breeding female *Cyld*/*Hdac6* double heterozygous (DHZ) mice (i.e., *Cyld* +/−, *Hdac6* +/−) with male *Cyld* heterozygous (CHZ) mice (i.e., *Cyld* +/−, *Hdac6* +/Y) (Figure 1A). The male *Cyld*/*Hdac6* DKO mice were viable and phenotypically normal, and showed no obvious variances in weight or behavior compared with their wild-type (WT) littermates. Male second-generation mice, including WT (i.e., *Cyld* +/+, *Hdac6* +/Y), CKO (i.e., *Cyld* −/−, *Hdac6* +/Y), HKO (i.e., *Cyld* +/+, *Hdac6* −/Y), and DKO mice, were selected for subsequent experiments due to the nature of the study, which included examination of sperm flagella.

To confirm the status of *Cyld* and *Hdac6* genes, we performed genotyping analysis for the first-generation (Figure 1B) and second-generation mice (Figure 1C). PCR analysis of mouse tail DNA with *Cyld*-specific primers produced a 200-bp band for WT and HKO mice, a 250-bp band for CKO and DKO mice, and both bands for DHZ and CHZ mice (Figure 1B and 1C). Similarly, PCR analysis with *Hdac6*-specific primers produced a 300-bp band for WT and CKO mice, a 250-bp band for HKO, DKO, and CHZ mice, and both bands for DHZ mice (Figure 1B and 1C). Western blot analysis of mouse liver lysates with antibodies specific for CYLD and HDAC6 confirmed that CYLD was not expressed in CKO mice, HDAC6 not expressed in HKO mice, and neither protein expressed in DKO mice (Figure 1D).

### Sperm flagellar defects induced by loss of *Cyld* are partially rescued by deletion of *Hdac6*

**Figure 2:**
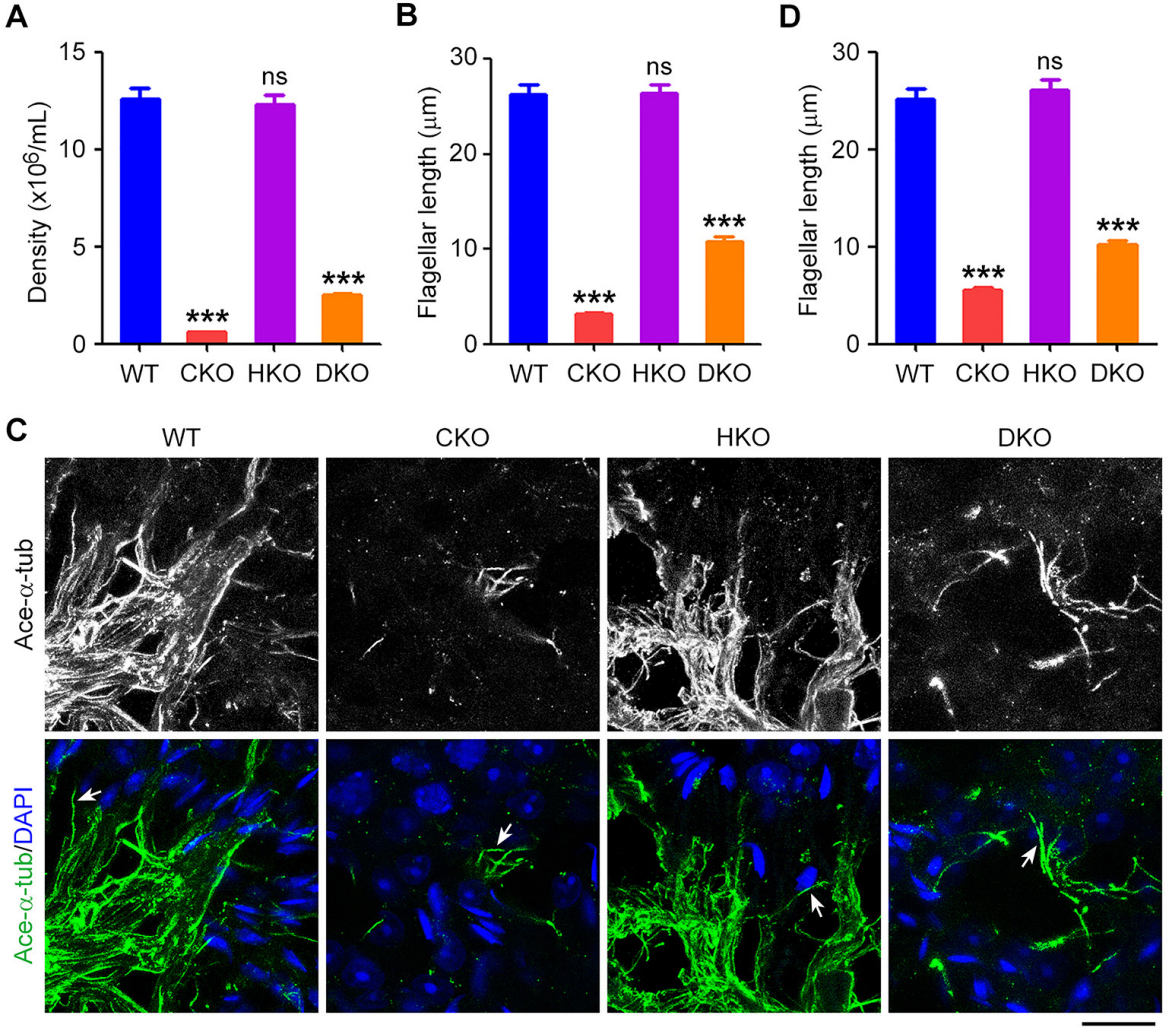
Sperm flagellar defects induced by loss of *Cyld* are partially rescued by deletion of *Hdac6* **A.** and **B.** Quantification of the density (A) and flagellar length (B) of sperm isolated from WT, CKO, HKO, and DKO mice. **C.** Immunofluorescence images of sperm flagella in the testis of mice, stained with acetylated α-tubulin (ace-α-tub) antibody and DAPI. Arrows indicate representative flagella. Scale bar, 15 μm. **D.** Experiments were performed as in C, and the flagellar length was quantified. ***P < 0.001; ns, not significant. Data are represented as mean ± SEM.

We examined sperm isolated from the epididymides of WT, CKO, HKO, and DKO mice. Consistent with our previous results [13], CKO mice produced impaired sperm, for which both the density of sperm and the length of sperm flagella were reduced (Figure 2A and 2B). In agreement with the previously reported effects of HDAC6 inhibitors and HDAC6 siRNAs on ciliogenesis [25], deletion of *Hdac6* did not significantly affect the density of sperm or the length of sperm flagella (Figure 2A and 2B). However, the sperm density and flagellar defects induced by loss of *Cyld* were partially restored in DKO mice (Figure 2A and 2B). We next analyzed sperm flagella in the testis by immunofluorescence staining with an antibody directed against acetylated α-tubulin, a well-characterized ciliary marker. Similar to results for isolated sperm, we found that the flagellar length was also partially rescued in DKO mice (Figure 2C and 2D). Together, these results suggest that the flagellar defects induced by loss of *Cyld* are partially rescued by deletion of *Hdac6*.

### *Cyld*/*Hdac6* DKO mice are protected from ciliary defects in the tracheal epithelium

**Figure 3:**
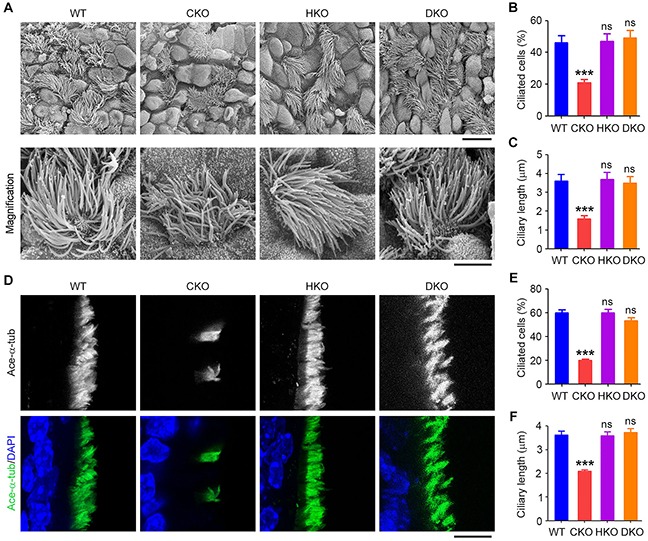
*Cyld*/*Hdac6* DKO mice are protected from ciliary defects in the tracheal epithelium **A.** Scanning electron microscopy images of cilia in WT, CKO, HKO, and DKO mouse tracheal epithelia. Scale bars, 3 μm. **B.** and **C.** Experiments were performed as in A, and the percentage of ciliated cells (B) and ciliary length (C) were quantified. **D.** Immunofluorescence images of tracheal epithelial cilia in WT, CKO, HKO, and DKO mice, stained with acetylated α-tubulin (ace-α-tub) antibody and DAPI. Scale bar, 5 μm. **E.** and **F.** Experiments were performed as in D, and the percentage of ciliated cells (E) and ciliary length (F) were quantified. ***P < 0.001; ns, not significant. Data are represented as mean ± SEM.

To investigate whether ciliary defects in the trachea caused by loss of *Cyld* could be rescued in *Cyld*/*Hdac6* DKO mice, scanning electron microscopy was performed to examine the tracheal surface epithelium of WT, CKO, HKO, and DKO mice. We found that CKO mice exhibited reductions in the percentage of ciliated cells and ciliary length, while HKO mice showed no significant ciliary defects compared with WT mice (Figures 3A–3C). In DKO mice, the percentage of ciliated cells and the length of cilia were significantly increased compared with CKO mice (Figures 3A–3C). Similar results were obtained by immunofluorescence staining of cilia in mouse trachea (Figures 3D–3F). These results indicate that tracheal motile ciliary defects induced by loss of *Cyld* are significantly rescued in DKO mice.

### *Cyld*/*Hdac6* DKO abrogates *Cyld* loss-induced ciliary defects in the kidney

**Figure 4:**
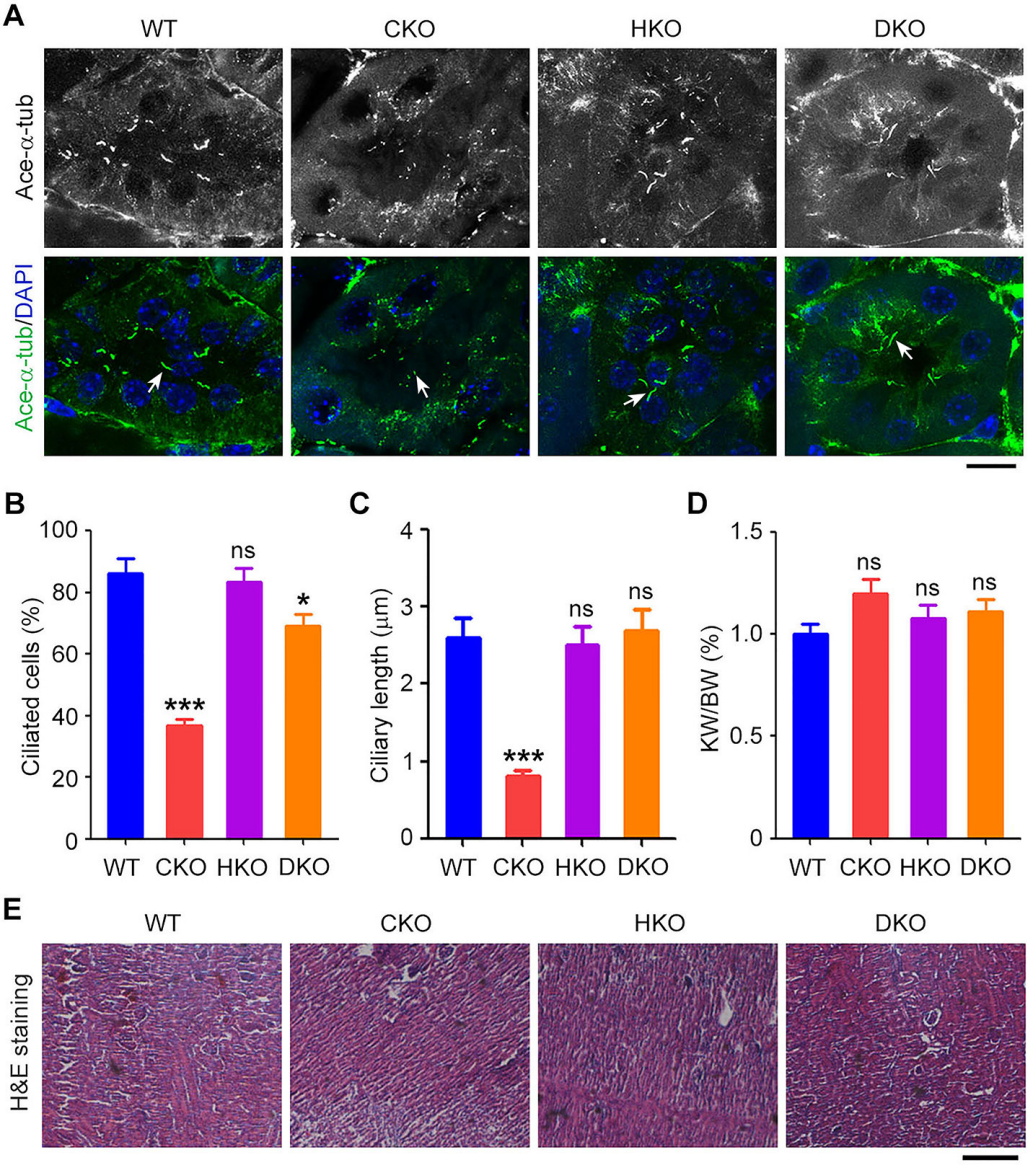
*Cyld*/*Hdac6* DKO abrogates *Cyld* loss-induced ciliary defects in the kidney **A.** Immunofluorescence images of renal cilia in WT, CKO, HKO, and DKO mice, stained with acetylated α-tubulin (ace-α-tub) antibody and DAPI. Arrows indicate representative cilia. Scale bar, 10 μm. **B.** and **C.** Experiments were performed as in A, and the percentage of ciliated cells (B) and ciliary length (C) were quantified. **D.** Statistical analysis of the kidney weight (KW)/body weight (BW) ratio for WT, CKO, HKO, and DKO mice. **E.** Images of hematoxylin/eosin (H & E) stained renal tissues of WT, CKO, HKO, and DKO mice. Scale bar, 10 μm. *P < 0.05, ***P < 0.001; ns, not significant. Data are represented as mean ± SEM.

We then examined whether renal ciliary defects caused by loss of *Cyld* are rescued by deletion of *Hdac6*. The primary cilia in the collecting ducts of the kidney were stained with acetylated α-tubulin antibody. We found that CKO mice exhibited reduced percentage of ciliated cells and decreased ciliary length, while deletion of *Hdac6* did not significantly affect cilia compared with WT mice (Figures 4A–4C). However, in DKO mice, ciliary defects induced by loss of *Cyld* in the kidney were efficiently rescued (Figures 4A–4C). These data suggest that DKO dramatically ablated the effect of *Cyld* loss on cilia.

Since PKD has been strongly associated with ciliary defects in the kidney [30], we sought to investigate whether the CYLD/HDAC6 axis contributes to this disease. We first examined the kidney weight/body weight ratio (KW/BW) of WT, CKO, HKO, and DKO mice and found no significant difference among these mice (Figure 4D). We then performed hematoxylin and eosin (H & E) staining of kidneys from WT, CKO, HKO, and DKO mice. We found that, similar to WT mice, CKO, HKO, and DKO mice did not exhibit obvious cysts in the kidney (Figure 4E). These data suggest that ciliary defects in the kidney do not always precipitate PKD.

### *Cyld* loss-induced ciliary defects in mouse embryonic fibroblasts (MEFs) are significantly rescued by deletion of *Hdac6*

**Figure 5:**
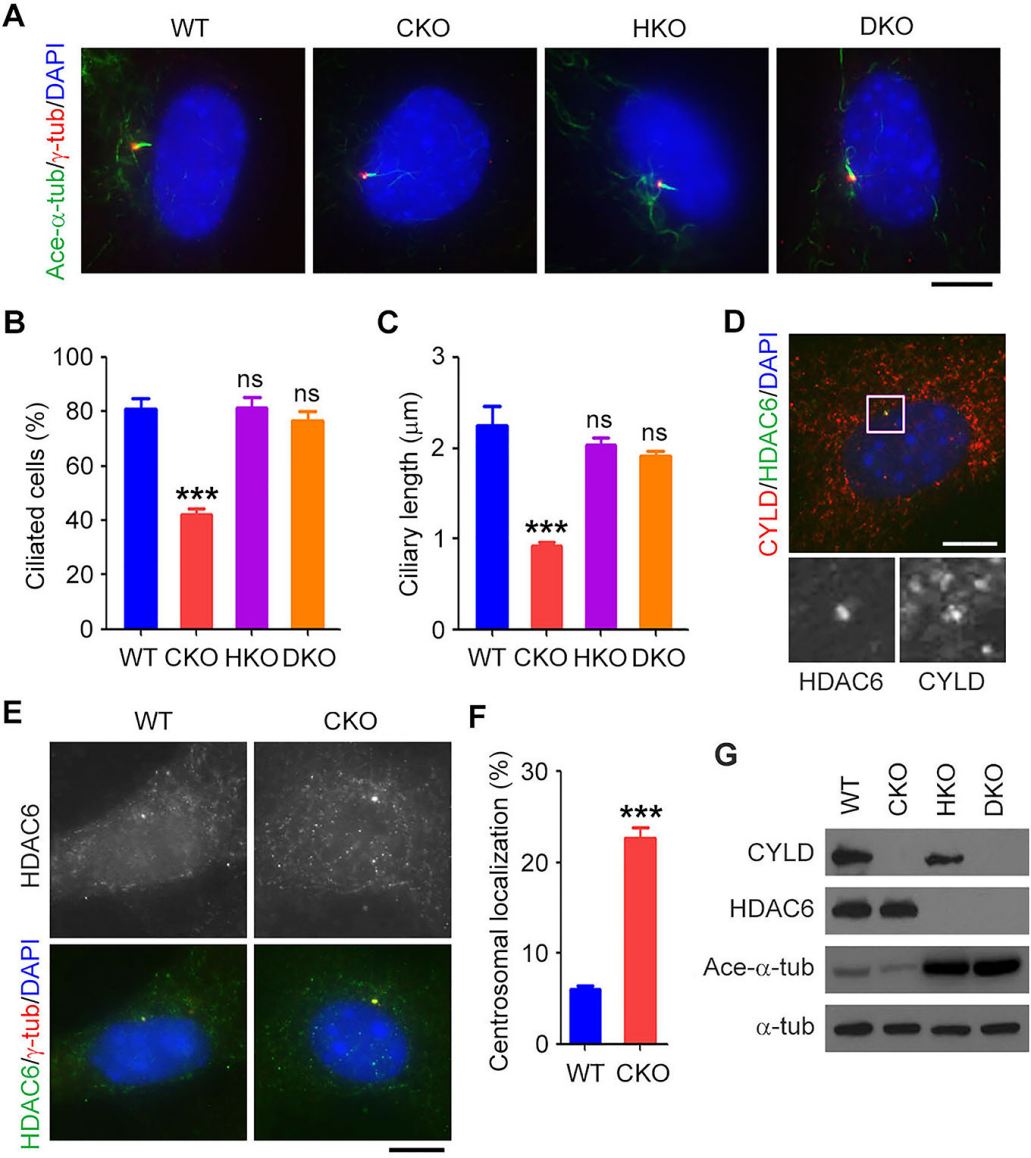
*Cyld* loss-induced ciliary defects in mouse embryonic fibroblasts (MEFs) are significantly rescued by deletion of *Hdac6* **A.** Immunofluorescence images of WT, CKO, HKO, and DKO MEFs serum-starved for 48 hours and stained with acetylated α-tubulin (ace-α-tub) and γ-tubulin (γ-tub) antibodies and DAPI. Scale bar, 8 μm. **B.** and **C.** Experiments were performed as in A, and the percentage of ciliated cells (B) and ciliary length (C) were quantified. **D.** Immunofluorescence images of WT MEFs serum-starved for 48 hours and stained with CYLD and HDAC6 antibodies and DAPI. The boxed area is shown in a higher magnification at the bottom. Scale bar, 8 μm. **E.** Immunofluorescence images of WT and CKO MEFs serum-starved for 48 hours and stained with HDAC6 and γ-tubulin (γ-tub) antibodies and DAPI. Scale bar, 8 μm. **F.** Experiments were performed as in E, and the percentage of HDAC6 localized at the centrosome/basal body was quantified. **G.** Western blot analysis of CYLD, HDAC6, acetylated α-tubulin (ace-α-tub), and α-tubulin (α-tub) in lysates from WT, CKO, HKO, and DKO MEFs. ***P < 0.001; ns, not significant. Data are represented as mean ± SEM.

The next question then is whether *Cyld* loss-induced ciliary defects could be rescued by deletion of *Hdac6 in vitro*. To answer this question, we investigated WT, CKO, HKO, and DKO MEFs. To induce ciliary formation, MEFs were cultured in serum-free medium for 48 hours. We found that both the percentage of ciliated cells and the length of cilia were dramatically reduced in CKO MEFs and that deletion of *Hdac6* did not affect cilia compared to WT MEFs (Figures 5A–5C). However, the ciliary defects induced by loss of *Cyld* were significantly restored in DKO MEFs (Figures 5A–5C).

To explore the molecular mechanism pertaining to the function of HDAC6 in the ciliary action of CYLD, MEFs were cultured in serum-free medium for 48 hours and double stained with CYLD and HDAC6 antibodies. We found that in a population of MEFs where HDAC6 were enriched at the centrosome/basal body, CYLD was also enriched in this region (Figure 5D). We next compared the localization of HDAC6 in WT and CKO MEFs. We found that loss of *Cyld* dramatically increased the intensity of HDAC6 localized at the centrosome/basal body (Figure 5E and 5F). In addition, simultaneous deletion of *Hdac6* significantly reversed the reduction of α-tubulin acetylation observed in CKO MEFs (Figure 5G). Together with previous findings [13], these results indicate that deletion of *Hdac6* in CKO mice significantly reverses *Cyld* loss-induced ciliary defects by acting on the localization and activity of HDAC6.

### Sonic hedgehog (Shh) signaling is restored in *Cyld*/*Hdac6* DKO MEFs

**Figure 6:**
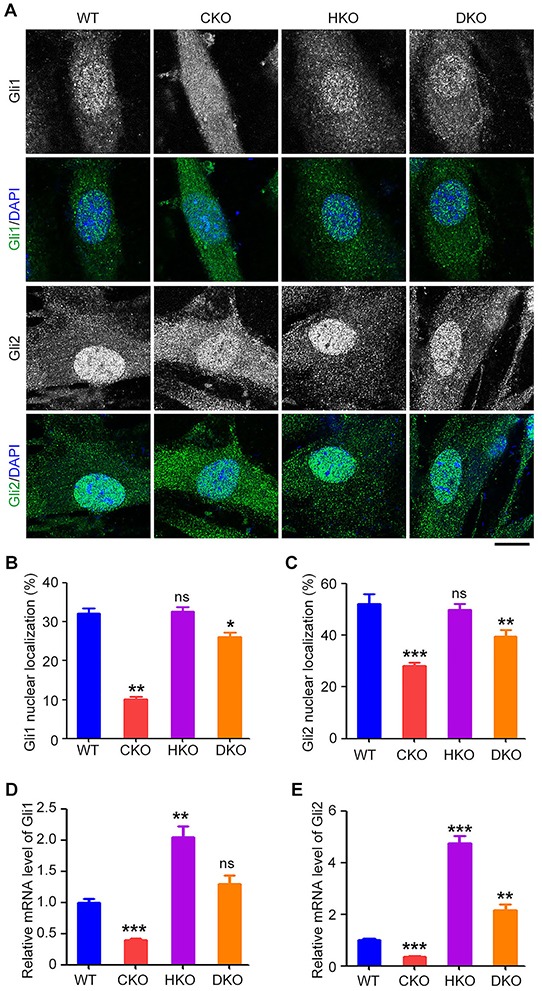
Sonic hedgehog (Shh) signaling is restored in *Cyld*/*Hdac6* DKO MEFs **A.** Immunofluorescence images of WT, CKO, HKO, and DKO MEFs serum-starved for 24 hours, treated with conditioned serum-free medium containing the amino-terminal domain of Shh (ShhN) for 24 hours, and stained with Gli1 or Gli2 antibody and DAPI. Scale bar, 10 μm. **B.** and **C.** Experiments were performed as in A, and the percentage of Gli1 (B) and Gli2 (C) localized in the nucleus was quantified. **D.** and **E.** Real-time quantitative RT-PCR analysis of the mRNA levels of Gli1 (D) and Gli2 (E) in WT, CKO, HKO, and DKO MEFs serum-starved for 24 hours and treated with ShhN-conditioned serum-free medium for 24 hours. *P < 0.05, **P < 0.01, ***P < 0.001; ns, not significant. Data are represented as mean ± SEM.

Because the CYLD/HDAC6 axis is involved in ciliary homeostasis [13], and ciliary assembly is required for the Shh signaling [31], we next assessed the Shh pathway in WT, CKO, HKO, and DKO MEFs. MEFs were cultured in serum-free medium for 24 hours, followed by treatment with conditioned serum-free medium containing the amino-terminal domain of Shh (ShhN) for 24 hours. Cells were then stained with antibodies directed against Gli1 and Gli2, two key transcriptional regulators of the Shh signaling [31]. We found that loss of *Cyld* dramatically reduced the proportion of Gli1 and Gli2 in the nucleus, but deletion of *Hdac6* did not significantly affect nuclear localization of these two proteins (Figures 6A–6C). In contrast, in DKO MEFs, the impaired nuclear localization of Gli1 and Gli2 was significantly restored (Figures 6A–6C). We also analyzed the mRNA levels of Gli1 and Gli2. In CKO MEFs, the mRNA levels of these two proteins were reduced, and in HKO MEFs, their mRNA levels were up-regulated compared to WT MEFs (Figure 6D and 6E). In DKO MEFs, the reduced mRNA levels induced by loss of *Cyld* were substantially reversed (Figure 6D and 6E). These data indicate that the impaired Shh signaling in CKO MEFs is efficiently restored by simultaneous deletion of *Hdac6*.

## DISCUSSION

HDAC6 is a cytoplasmic member of the HDAC family. Unlike most other HDACs, HDAC6 primarily deacetylates non-histone proteins and thereby plays important roles in a variety of cellular processes, such as cell signaling, cell motility, and cell-cell interactions [32–43]. Recently, there is accumulating evidence that HDAC6 is a critical regulator of ciliary homeostasis [15–26]. α-Tubulin and cortactin, two substrates of the deacetylase activity of HDAC6, have been identified as the major downstream elements that mediate the ciliary function of HDAC6 [25]. In addition, HDAC6 has been shown to mediate the actions of other cilium-associated proteins, such as Aurora A, death inducer obliterator 3 (Dido3), and Polo-like kinase 1 (Plk1) [15–26]. While the molecular mechanisms by which these proteins regulate the ciliary role of HDAC6 are elusive, our data demonstrate that HDAC6 is enriched at the centrosome/basal body and that this localization is enhanced by the loss of *Cyld*. These findings suggest that regulation of HDAC6 localization or activity at the centrosome/basal body may represent a critical mechanism controlling the functions of HDAC6 in ciliary homeostasis.

CYLD was initially identified as a tumor suppressor and has recently been identified as an important ciliary regulator, as both CKO mice and transgenic mice carrying truncations in the carboxyl-terminal deubiquitinase domain of CYLD exhibit ciliary defects [13, 14]. Our data show that ciliary defects resulting from loss of *Cyld* are efficiently reversed by simultaneous deletion of *Hdac6* in organs ranging from the trachea to the kidney, as well as at the cellular level in MEFs. CKO mice have impaired flagella, and since a properly functioning flagellum is essential for sperm motility, these mice are infertile. Interestingly, although the sperm flagellar defects induced by loss of *Cyld* are partially restored in *Cyld*/*Hdac6* DKO mice, these mice remain infertile. However, this is not surprising given that spermatogenesis is a tightly controlled event, and the formation of flagella is only one element of this process. Further studies are warranted to examine the functionality of the flagella in DKO mice, such as sperm motility.

Over the past decade, HDAC6 has emerged as an attractive target for the treatment of a variety of diseases, including neurodegenerative disorders and cancer [44–46]. Unlike many pan-HDAC inhibitors, HDAC6-targeted therapies exhibit no obvious side effects. For example, tubastain A, a selective inhibitor of HDAC6, have been shown to protect mice from diverse ciliary defects without causing toxicity to normal tissues [13]. Tubastain A also restores cilia in cholangiocarcinoma cells and induces a significant decrease in tumor growth in a cholangiocarcinoma animal model without causing obvious side effects [46]. The present study demonstrates that deletion of *Hdac6* rescues ciliary defects induced by *Cyld* loss in the testis, trachea, and kidney without affecting other organs. These data, together with previous findings that small-molecule compounds inhibiting HDAC6 restore ciliary defects in nephronophthisis and chronic obstructive pulmonary disease [18, 26], suggest the potential therapeutic value of HDAC6-selective inhibitors in ciliopathies.

## MATERIALS AND METHODS

### Ethics statement

Investigation has been conducted in accordance with the ethical standards according to the Declaration of Helsinki and the national and international guidelines, and has been approved by the authors' institutional review board.

### Mice

CKO and HKO mice were generated and genotyped as described previously [47, 48]. *Cyld*/*Hdac6* DKO mice were generated and genotyped as described in Figure 1. Six mice were used for each experimental group.

### Cell culture

MEFs were isolated from E13.5 mouse embryos as described previously [13]. All MEFs were cultured in Dulbecco's Modified Essential Medium supplemented with 10% fetal bovine serum at 37°C in a humidified atmosphere containing 5% CO_2_. To induce ciliary formation, MEFs were cultured in serum-free medium for 48 hours.

### Antibodies

Antibodies against acetylated α-tubulin, γ-tubulin, β-actin (Sigma-Aldrich), CYLD, Gli1 (Santa Cruz Biotechnology), α-tubulin and Gli2 (Abcam), and HDAC6 (Abgent) were purchased from the indicated sources. Fluorescein- and rhodamine-conjugated secondary antibodies were purchased from Jackson ImmunoResearch Laboratories, and horseradish peroxidase-conjugated secondary antibodies were purchased from Santa Cruz Biotechnology.

### Immunofluorescence microscopy

Mouse tissues were fixed in 4% paraformaldehyde, embedded, and snap-frozen in liquid nitrogen prior to sectioning as described [9]. Tissue sections or MEFs grown on glass coverslips were fixed with 4% paraformaldehyde for 20 minutes and permeabilized in 0.5% Triton X-100 in PBS for 20 minutes at room temperature. They were then blocked with a buffer containing 2% bovine serum albumin in PBS for 1 hour at room temperature. Sections were then incubated with indicated primary antibodies overnight at 4°C and with appropriate secondary antibodies for 1 hour at room temperature, followed by staining with DAPI (Sigma-Aldrich). Finally, the coverslips were mounted with anti-fade medium and examined with a TCS SP5 confocal microscope (Leica). The percentage of ciliated cells and ciliary length were measured using the ImageJ software (National Institutes of Health, USA). The fluorescence intensity of proteins at specific regions was quantified as described previously [49].

### Scanning electron microscopy

Mouse tracheal samples were isolated and fixed with 2.5% (vol/vol) glutaraldehyde in 0.1 M sodium cacodylate as described previously [13]. Samples were post-fixed in 1% (wt/vol) osmium tetroxide for 2 hours, dehydrated with graded ethanol, and dried in a critical point dryer. The samples were gold-coated and examined with a QUANTA 200 scanning electron microscope.

### Sperm analysis

Sperm were extracted from cauda epididymides, and the length of flagella were measured with the ImageJ software as described previously [13].

### Histological analysis

Kidneys were harvested immediately after mice were sacrificed, and kidney/body weights were recorded. Samples were fixed in 10% formalin overnight at 4°C and embedded in paraffin. Tissue sections (4 μm) were cut, deparaffinized, rehydrated with graded ethanol, and stained with hematoxylin and eosin (Sigma-Aldrich). Slides were visualized with a Leica DM3000 camera (Leica).

### Western blotting

Proteins were resolved by SDS-PAGE and transferred to polyvinylidene difluoride membranes (Millipore). The membranes were blocked with 5% non-fat milk in Tris-buffered saline containing 0.2% Tween 20, then incubated with primary antibodies overnight at 4°C, followed by incubation with appropriate secondary antibodies for 45 minutes at room temperature. Specific proteins were visualized with enhanced chemiluminescence detection reagents according to the manufacturer's instructions (Thermo Fisher Scientific).

### Quantitative real-time RT-PCR

Total RNA was isolated from MEFs using the TRIzol reagent (Invitrogen). Quantitative real-time RT-PCR was performed using the SYBR Premix Ex Taq (Perfect Real Time) reagent (Takara) as described previously [50, 51]. β-Actin was used as a control to normalize the reading in each sample.

### Statistics

Analysis of statistical significance was performed by the Student's t-test for comparison between two groups and by the ANOVA test for multiple comparisons.
